# Online decision tools for personalized survival prediction and treatment optimization in elderly patients with lung squamous cell carcinoma: a retrospective cohort study

**DOI:** 10.1186/s12885-023-11309-z

**Published:** 2023-09-29

**Authors:** Chen-ye Shao, Jing Luo, Sheng Ju, Chu-ling Li, Cheng Ding, Jun Chen, Xiao-long Liu, Jun Zhao, Li-qin Yang

**Affiliations:** 1 Department of Thoracic Surgery, The First Affiliated Hospital of Soochow University, 899 Pinghai Road, Gusu District, Suzhou, 215006 China; 2 Institute of Thoracic Surgery, The First Affiliated Hospital of Soochow University, 899 Pinghai Road, Gusu District, Suzhou, 215006 China; 3 Department of Respiratory Medicine, Jinling Hospital, Nanjing Medical University, Nanjing, China; 4 Department of Respiratory Medicine, Jinling Hospital Medical School of Nanjing University, Nanjing, China; 5 Department of Cardiothoracic Surgery, Jinling Hospital, Medical School of Nanjing University, 305 Zhongshan East Road, Nanjing, 210002 Jiangsu China

**Keywords:** Adjuvant therapy, Nomogram, Lung Squamous Cell Carcinoma

## Abstract

**Background:**

Despite major advances in cancer therapeutics, the therapeutic options of Lung Squamous Cell Carcinoma (LSCC)-specific remain limited. Furthermore, the current staging system is imperfect for defining a prognosis and guiding treatment due to its simplicity and heterogeneity. We sought to develop prognostic decision tools for individualized survival prediction and treatment optimization in elderly patients with LSCC.

**Methods:**

Clinical data of 4564 patients (stageIB-IIIB) diagnosed from 2010 to 2015 were extracted from the Surveillance, Epidemiology, and End Results (SEER) database for prognostic nomograms development. The proposed models were externally validated using a separate group consisting of 1299 patients (stage IB-IIIB) diagnosed from 2012–2015 in China. The prognostic performance was measured using the concordance index (C-index), calibration curves, the average time-dependent area under the receiver operator characteristic curves (AUC), and decision curve analysis.

**Results:**

Eleven candidate prognostic variables were identified by the univariable and multivariable Cox regression analysis. The calibration curves showed satisfactory agreement between the actual and nomogram-estimated Lung Cancer-Specific Survival (LCSS) rates. By calculating the c-indices and average AUC, our nomograms presented a higher prognostic accuracy than the current staging system. Clinical usefulness was revealed by the decision curve analysis. User-friendly online decision tools integrating proposed nomograms were created to estimate survival for patients with different treatment regimens.

**Conclusions:**

The decision tools for individualized survival prediction and treatment optimization might facilitate clinicians with decision-making, medical teaching, and experimental design. Online tools are expected to be integrated into clinical practice by using the freely available website (https://loyal-brand-611803.framer.app/).

**Supplementary Information:**

The online version contains supplementary material available at 10.1186/s12885-023-11309-z.

## Introduction

Lung cancer remains the leading cause of cancer-related death worldwide, with an estimated 1.79 million deaths per year [1]. Approximately 85% of total diagnoses are non-small cell lung cancer (NSCLC), of which lung adenocarcinoma (LUAD) and lung squamous cell carcinoma (LSCC) are the most common subtypes. While the ongoing discovery of novel oncogenic mutations and development of new targeted therapies have demonstrated efficacy in prolonging the progression-free survival (PFS) and overall survival (OS) in LUAD patients, therapies aimed at these pathways performed poorly among LSCC [2, 3]. The advent of immune checkpoint inhibitor (ICI) therapy provides a viable treatment option for LSCC [4]. Despite showing significant benefits, immunotherapies were plagued by a relatively low response rate and a high rate of immunotherapy-related adverse effects in some cases. Furthermore, evidence suggests that adoption of ICIs has been linked to significant benefits for younger patients with NSCLC. Survival benefits in the old age group, on the other hand, were less substantial [5]. Thus, the current treatment options for elderly patients with advanced LSCC remain limited. For these patients, postoperative cisplatin-based adjuvant therapy still needs to be actively considered.

The pathological staging system is a fundamental cornerstone of postoperative surveillance and clinical management. The current 8^th^ edition of the staging system for NSCLC includes 3 components: extent of the primary tumor (T), lymph node involvement (N, which is determined by the anatomical regions of metastatic lymph nodes) and distant metastasis (M) [6]. Nevertheless, it remains imperfect to rely solely on this staging system for individual survival prediction and treatment decision-making. This might be explained for at least two reasons listed as follows: 1. the survival rates differ significantly within the same stage subgroup [7–9]; 2. Other potential prognostic factors were omitted, such as grade, the number of metastatic lymph nodes [10–12], age [13, 14], etc. While several clinical models have been developed to improve risk stratification and survival prediction in NSCLC [15–17], few exist for the analysis of optimal postoperative treatment regimens (without adjuvant therapy [AT], adjuvant chemotherapy [ACT], adjuvant chemoradiotherapy [ACRT]) in elderly patients with LSCC. To address this gap, we sought to establish and validate online decision tools for individualized prognosis prediction and postoperative treatment guidance by incorporating the TNM staging system and other critical prognostic factors. We present this manuscript following the TRIPOD reporting checklist.

## Methods

### Data extraction and patient population

**Fig. 1:**
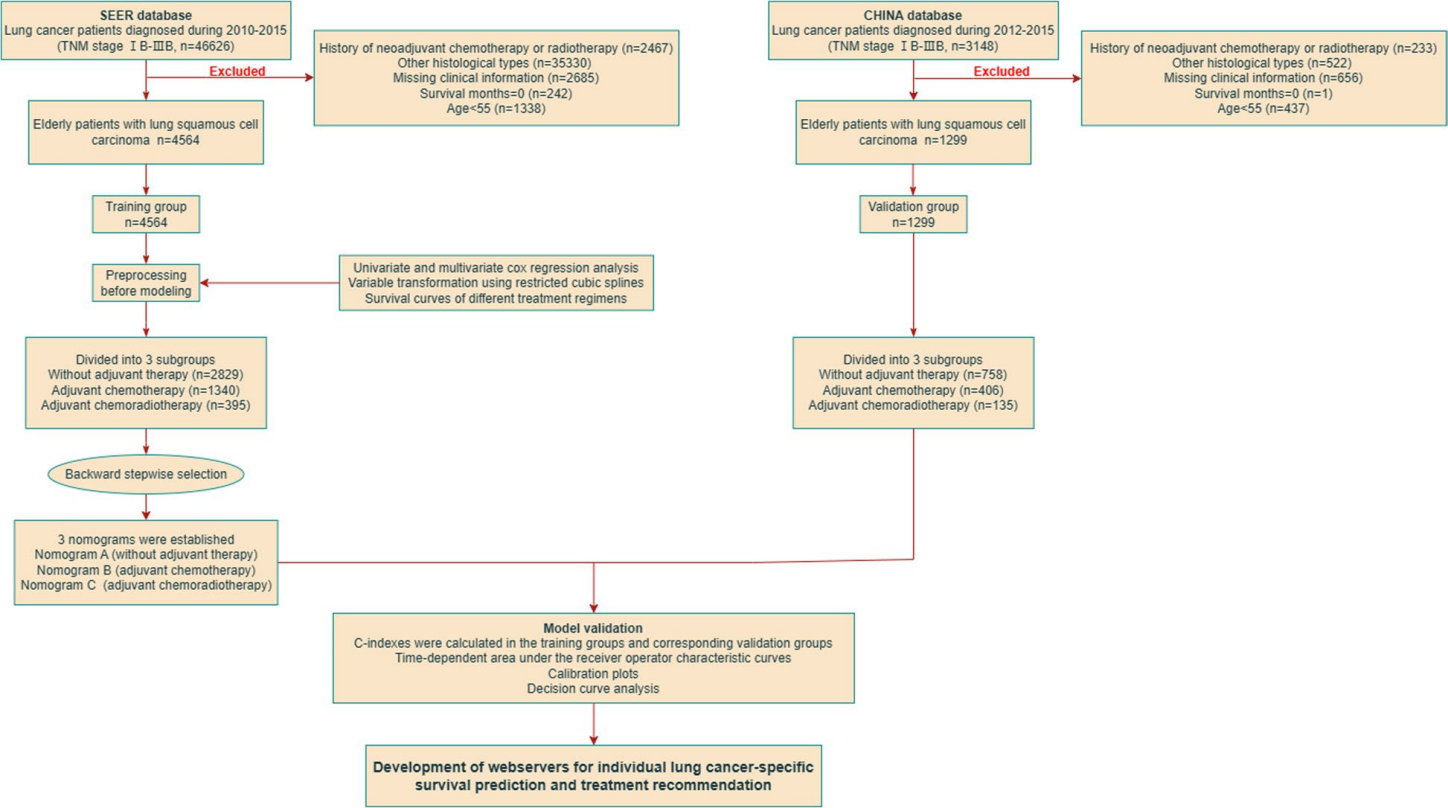
Flowchart of patient screening and study procedure

This retrospective observational study was approved by the institutional review board and the research ethics committee of the First Affiliated Hospital of Soochow University (No. 2022–633). Patients from the Surveillance, Epidemiology, and End Results (SEER) database were included based on the following criteria: (1) primary LSCC confirmed by pathology (TNM stage: IB-IIIB); (2) patients diagnosed between January 2010 and December 2015; (3) regional nodes examined ≥ 1 (to obtain a relatively accurate number of positive lymph nodes); (4) age ≥ 55 at diagnosis; (5) underwent surgery. Patients were excluded if they met the following criteria: (1) a history of neoadjuvant chemotherapy or radiotherapy; (2) missing clinicopathology data; (3) perioperative death. A total of 4564 cases extracted from the SEER database were enrolled as the training group. The citation for the selected database: Surveillance, Epidemiology, and End Results (SEER) Program (https://seer.cancer.gov/) [18]. Variables for nomogram model development included age at diagnosis, race, sex, primary site, grade, separate tumor nodules, pleural invasion record, T stage, N stage, eighth TNM stage, surgical type, number of positive lymph nodes and adjuvant therapy information. An external validation group comprised 1299 patients from 2 medical institutions in CHINA: the First Affiliated Hospital of Soochow University (*n* = 659, from September 2012 to May 2015) and Jinling Hospital of Nanjing Medical University (*n* = 640, from December 2012 to November 2015). The screening criteria of the validation group were consistent with the training group, the related details are reported in Fig. 1. The outcome of interest in this study was Lung Cancer-Specific Survival (LCSS), which was defined as the interval between the time of diagnosis and lung cancer-related death. Individuals who die of causes other than the specified cause are considered to be censored. Written informed consent was waived due to retrospective anonymous analysis of data.

### Prognostic nomograms development

Univariable Cox regression analyses were performed to screen candidate prognostic variables in the training group (*n* = 4564). Statistically significant variables (*P* < 0.05) were further analyzed in multivariable Cox regression model. We used restricted cubic splines to flexibly model and visualize the associations between age and cancer-specific survival based on Cox proportional hazard models. The optimal number of knots between 3 and 7 was chosen based on the Akaike Information Criterion (AIC). The AIC value is commonly used to balance model complexity and goodness of fit. A lower AIC value indicates that the model achieves sufficient goodness of fit with fewer parameters. Therefore, model with the smallest AIC value was finally chosen to reduce the risk of overfitting. The continuous variable (age at diagnosis) would be categorized if non-linearity is detected. Survival curves of different groups of adjuvant therapy (without AT, ACT, and ACRT) were plotted within each subgroup stratified by the eighth staging system using the Kaplan–Meier estimates and were compared using the log-rank test. Patients of training groups were also divided into 3 subgroups according to the different treatment regimens. Subsequently, in different subgroups, a backward stepwise selection based on AIC values was performed for significant variables identified in univariable or multivariable analysis. Cox proportional hazards modeling was used for multivariable regression analysis to develop the survival prediction model.

### Nomograms validation and evaluation

Internal validation was performed using Bootstrapping with 500 resampling method and external validation was performed using an external validation group. Discrimination and calibration of the proposed nomograms were evaluated by calculating the concordance index (C-index) and plotting calibration curves in the training groups and corresponding validation groups. Time-dependent receiver operating characteristic (ROC) curves were also plotted [19]. The area under the curve (AUC) of time-dependent ROC was calculated every 2 months from the 1^st^ to the 60^th^ month. We used decision curve analysis (DCA) to estimate the net benefits at different threshold probabilities to assess the clinical usefulness of the proposed nomograms. C-indices and time-dependent ROC curves were also employed to compare the performance between the nomograms and the 8^th^ TNM staging system.

### Online prognostic tools establishment

To facilitate clinicians’ usage of the established nomograms, we created a user-friendly website for individualized survival prediction and postoperative treatment optimization in elderly patients with LSCC.

### Statistical analysis

The unpaired, χ^2^ test or the Fisher exact test was used to assess differences in distributions between the categorical variables studied. The Shapiro–Wilk test was used to assess normality of the continuous data. Based on the test results, appropriate statistical tests (t-test or Mann–Whitney test) were used for comparing the continuous variables. All statistical analyses were performed using R software (version 4.2.0, http://www.r-projiect.org/) and SPSS for Windows (version 23.0, IBM, New York, USA). A 2-sided *P* value < 0.05 was considered significant.

## Results

### Demographic and clinical characteristics

According to the inclusion and exclusion criteria (details of screening process were shown in Fig. 1), a total of 4564 patients were extracted from the SEER database and included as the training group. In the training group, 2247 deaths were observed during the follow-up time. The median follow-up was 31 months (IQR 9–49). The LCSS rates at 1, 3, and 5 years were 88.1%, 65.4%, and 52.4% (median survival time: 64 months). The external validation group comprised 1299 patients from the First Affiliated Hospital of Soochow University (*n* = 659) and Jinling Hospital of Nanjing Medical University (*n* = 640). In the validation group, there were 552 deaths observed during the follow-up time. The median follow-up was 28 months (IQR 10–47). The LCSS rates at 1, 3, and 5 years were 93.1%, 69.8%, and 59.1% (median survival time: 68 months). Detailed information of training group and validation group was illustrated in Table 1.

**Table 1:** Characteristics of training group and validation group

	Training group	Validation group
No. of cases	4564	1299
Year of diagnosis	2010–2015	2012–2015
Age at diagnosis (mean ± SD)	70.44 ± 7.58	65.08 ± 9.01
Race		
White	4001 (87.7%)	0 (0%)
Black	399 (8.7%)	0 (0%)
Asian	149 (3.3%)	1299 (100%)
Others	15 (0.3%)	0 (0%)
Sex		
Male	2899 (63.5%)	848 (65.3%)
Female	1665 (36.5%)	451 (34.7%)
Primary Site		
Main bronchus	67 (1.5%)	37 (2.8%)
Upper lobe	2524 (55.3%)	753 (58%)
Middle lobe	194 (4.3%)	55 (4.2%)
Lower lobe	1670 (36.6%)	424 (32.6%)
Overlapping lesion	109 (2.4%)	30 (2.3%)
Grade		
Unknown	126 (2.8%)	39 (3%)
Grade I	101 (2.2%)	36 (2.8%)
Grade II	1993 (43.7%)	625 (48.1%)
Grade III	2293 (50.2%)	587 (45.2%)
Grade IV	51 (1.1%)	12 (0.9%)
Separate tumor nodules		
None	4134 (90.6%)	1216 (93.6%)
Same lobe	313 (6.9%)	68 (5.2%)
Different lobe	103 (2.3%)	13 (1%)
Same and different lobes	14 (0.3%)	2 (0.2%)
Pleural invasion record		
PL0	2617 (57.3%)	769 (59.2%)
PL1 or PL2	1368 (30%)	409 (31.5%)
PL3	579 (12.7%)	121 (9.3%)
T stage		
T1a	45 (1%)	11 (0.8%)
T1b	111 (2.4%)	23 (1.8%)
T1c	149 (3.3%)	41 (3.2%)
T2a	2076 (45.5%)	789 (60.7%)
T2b	843 (18.5%)	145 (11.2%)
T3	1098 (24.1%)	226 (17.4%)
T4	242 (5.3%)	64 (4.9%)
N stage		
N0	2985 (65.4%)	876 (67.4%)
N1	1046 (22.9%)	295 (22.7%)
N2	516 (11.3%)	124 (9.5%)
N3	17 (0.4%)	4 (0.3%)
Eighth TNM stage		
IB	1528 (33.5%)	643 (49.5%)
IIA	590 (12.9%)	73 (5.6%)
IIB	1469 (32.2%)	342 (26.3%)
IIIA	806 (17.7%)	201 (15.5%)
IIIB	171 (3.7%)	40 (3.1%)
Surgical type		
Sub-lobar resection	417 (9.1%)	211 (16.2%)
Lobectomy	3696 (81%)	1005 (77.4%)
Peumonectomy	451 (9.9%)	83 (6.4%)
Number of positive lymph nodes (mean ± SD)	1.13 ± 5.39	0.97 ± 4.08
Adjuvant therapy		
Without adjuvant therapy	2829 (62%)	758 (58.4%)
Adjuvant chemotherapy	1340 (29.4%)	406 (31.3%)
Adjuvant chemoradiotherapy	395 (8.7%)	135 (10.4%)

Grade I, well differentiated; Grade II, moderately differentiated; Grade III, poorly differentiated; Grade IV, undifferentiated; PL0, tumor does not completely traverse the elastic layer of pleura; PL1 or PL2, invasion of visceral pleura present; PL3, tumor invades into or through the parietal pleura or chest wall; *SD* Standard deviation

### Development of prognostic nomograms in the training group

**Fig. 2:**
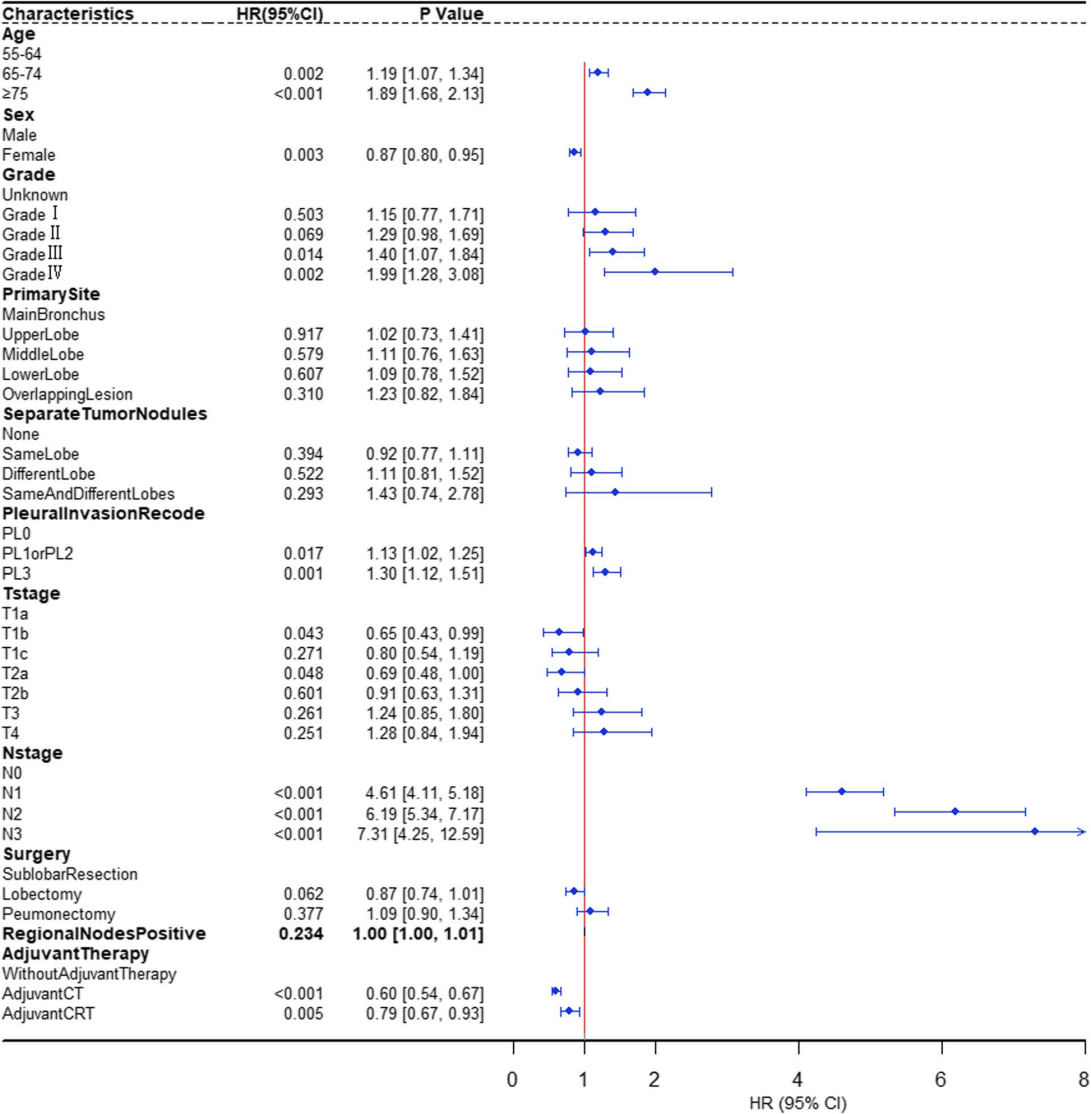
The forest plots showing the multivariable Regression Analysis of variables associated with Cancer-Specific Survival. Grade I, well differentiated; Grade II, moderately differentiated; Grade III, poorly differentiated; Grade IV, undifferentiated; PL0, tumor does not completely traverse the elastic layer of pleura; PL1 or PL2, invasion of visceral pleura present; PL3, tumor invades into or through the parietal pleura or chest wall; CT, chemotherapy; CRT, chemoradiotherapy

In univariable analysis, age, sex, primary site, grade, separate tumor nodules, pleural invasion record, T stage, N stage, Surgery, regional nodes positive, and adjuvant therapy were significantly associated with LCSS in the training group (all *P* < 0.05, Figure S1). Association between age and cancer-related mortality was depicted using the Restricted Cubic Spline Regression Model (Figure S2). The risk of cancer-specific mortality increased slowly until around 70 years of age at diagnosis and then started to increase rapidly afterward. Interestingly, our findings indicate that younger age may have a protective effect (hazard ratio, HR < 1) until approximately the age of 70. Subsequently, the HR exceeds 1, suggesting an increased risk of cancer-specific mortality. *P* for non-linearity < 0.001, which implies a nonlinear relationship between age and cancer-specific mortality. Thus, age was then transformed into categorical variable as follows: 55–64, 65–74, and ≥ 75. According to the consequences of the multivariable analysis, the following variables remained statistically significant: age, sex, grade, pleural invasion record, T stage, N stage, and adjuvant therapy (*P* < 0.05, Fig. 2). Survival curves were plotted to depict survival differences between groups stratified by different treatment regimens in the training group (Figure S3). Patients receiving certain treatment regimens had significantly better LCSS compared with those without adjuvant therapy was defined as survival benefit. For ACT, survival benefit was observed in stage IIA, stage IIB, stage IIIA, and stage IIIB. For ACRT, survival benefit was observed in stage IIIB.

**Fig. 3:**
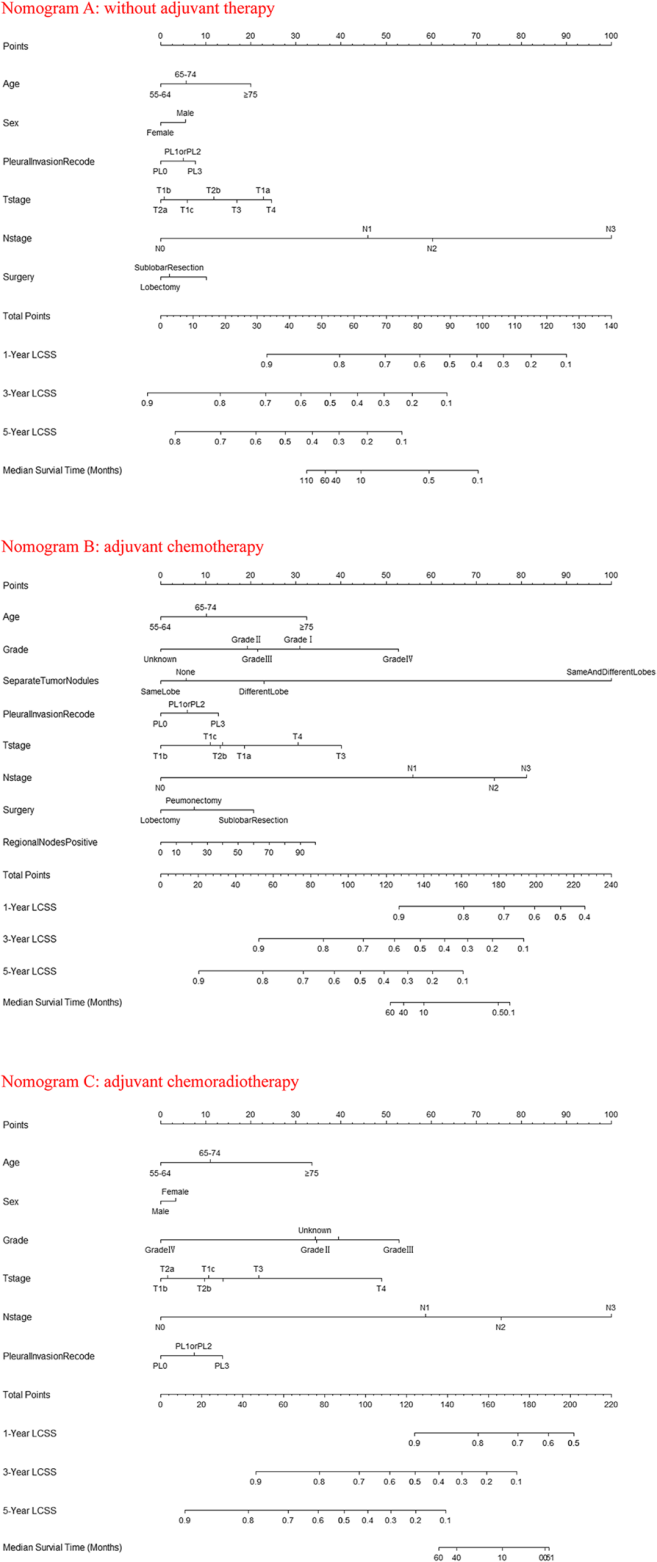
Nomograms for comparing expected Cancer-Specific Survival (CSS) with different postoperative treatment regimens (Nomogram A, without adjuvant therapy; Nomogram B, adjuvant chemotherapy; Nomogram C, adjuvant chemoradiotherapy). For an individual patient, use nomogram A, B, and C in sequence to calculate expected CSS with different regimens. Determine the optimal treatment regimen by comparing the 1-year, 3-year, and 5-year CSS rates between these 3 groups. Grade I, well differentiated; Grade II, moderately differentiated; Grade III, poorly differentiated; Grade IV, undifferentiated; PL0, tumor does not completely traverse the elastic layer of pleura; PL1 or PL2, invasion of visceral pleura present; PL3, tumor invades into or through the parietal pleura or chest wall. To utilize the nomogram, start by drawing a vertical line to the top points row to assign points for each variable. Next, sum up the points from each variable and draw a vertical line from the total points row to determine the 1-year survival, 3-year survival, 5-year survival, and median survival time (in months)

Thus, instead of including different treatment regimens into one same nomogram model, we divided the training group into 3 groups (without AT, *n* = 2829, ACT, *n* = 1340, and ACRT, *n* = 395) and constructed nomograms respectively. After stepwise selection to remove potential redundant variables based on Akaike Information Criterion, 3 nomograms were finally established: Nomogram A (without AT, 6 variables), Nomogram B (ACT, 8 variables), and Nomogram C (ACRT, 6 variables). The details of the established 3 nomograms are shown in Fig. 3. To determine the optimal treatment regimen, use nomogram A, B, and C in sequence to calculate expected LCSS, then compare the 1-year, 3-year, and 5-year LCSS rates between these 3 groups.

### Evaluation and validation of the proposed nomograms

As shown in Table 2, the discrimination of the proposed nomograms was superior to that of the TNM staging system in both the training groups (C-indices for LCSS estimates: without AT, 0.735 vs 0.693; ACT, 0.707 vs 0.662; ACRT, 0.718 vs 0.643; all *P* < 0.05) and validation groups (C-indices for LCSS estimates: without AT, 0.718 vs 0.658; ACT, 0.747 vs 0.705; ACRT, 0.701 vs 0.568; all *P* < 0.05). Similar results could be observed by calculating the time-dependent area under the receiver operator characteristic curves every 2 months from the 1^st^ to the 60^th^ month (Figure S4). The average AUCs of the proposed nomograms were higher than those of the TNM staging system in the training groups (without AT, 0.797 vs 0.745; ACT, 0.727 vs 0.665; ACRT, 0.84 vs 0.764), indicating the superior prognostic ability of these nomograms. Moreover, the calibration curves demonstrated good concordance between the nomogram-predicted and the actual 1-, 3-, and 5-year LCSS probability in the training groups (Figure S5) and validation groups (Figure S6). Decision curve analysis was performed to assess the net benefit of nomogram-assisted decisions at different threshold probabilities, compared with the net benefit of decisions made with the assumption that either all or no patient survive during the follow-up period [20]. The decision analysis curves plotted for 1-, 3-, and 5-year LCSS in the training group demonstrated the potential benefits and clinical utility using these nomograms (Figure S7).

**Table 2:** Prognostic performance of 3 proposed nomograms and the 8^th^ edition of the UICC/AJCC staging system in the training groups and corresponding validation groups

Model	Training group		Validation group	
	C-index (95% CI)	*P* value	C-index (95% CI)	*P* value
Without AT				
Proposed nomogram A	0.735 (0.723–0.748)	Reference	0.718 (0.689–0.747)	Reference
Eighth TNM stage	0.693 (0.679–0.707)	< .05	0.658 (0.629–0.687)	< .05
ACT				
Proposed nomogram B	0.707 (0.687–0.727)	Reference	0.747 (0.714–0.780)	Reference
Eighth TNM stage	0.662 (0.642–0.682)	< .05	0.705 (0.672–0.738)	< .05
ACRT				
Proposed nomogram C	0.718 (0.683–0.753)	Reference	0.701 (0.638- 0.764)	Reference
Eighth TNM stage	0.643(0.608–0.678)	< .05	0.568 (0.505–0.631)	< .05

*UICC* Union for International Cancer Control, *AJCC* American Joint Committee on Cancer, *C-index* Concordance index, *Without AT* Without adjuvant therapy, *ACT* Adjuvant chemotherapy, *ACRT* Adjuvant chemoradiotherapy, *TNM* Tumor, node, and metastasis, *p*-value indicates the difference in the C-indices

### Online decision tools

**Fig. 4:**
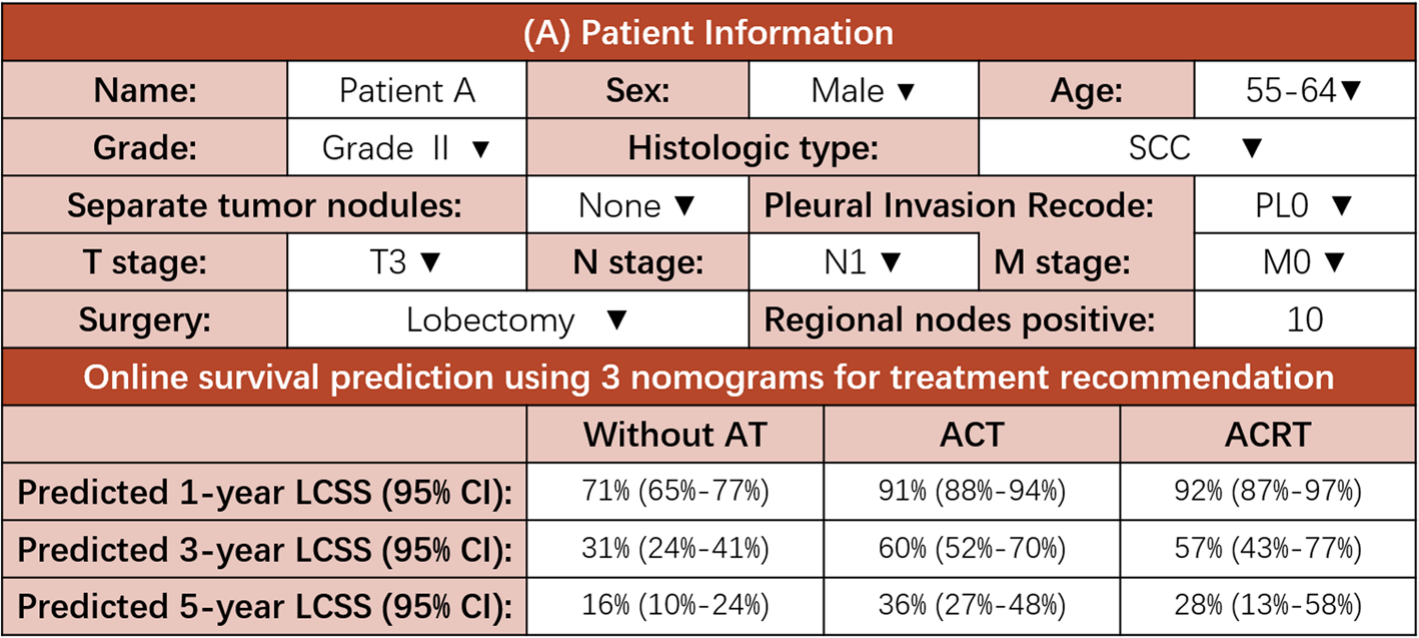
Decision tools for treatment optimization by using online nomograms A, B, and C in sequence. Clinicians could compare the 1-year, 3-year, and 5-year CSS rates between these 3 groups to assist in clinical decision-making. CSS, cancer-specific survival; Without AT, without adjuvant therapy; ACT, adjuvant chemotherapy; ACRT, adjuvant chemoradiotherapy

We further created online decision-making tools with two main functions (https://loyal-brand-611803.framer.app/). For individualized survival prediction, by entering the clinical information of the requested patient into the interface on the left side of the webpage, users could obtain the estimated Kaplan–Meier curve (top right) and the predicted survival probability (bottom right) of this case (Figure S8). The function of treatment optimization is implemented by using nomogram A, B, and C in sequence. Figure 4 displayed clinical information and predicted survival rates (using online decision tools above) of a hypothetical patient diagnosed with LSCC (T3N1M0, Stage III A). Detailed information of patient was shown in the diagram. For this patient, survival benefits (defined as patients receiving certain treatment regimens had significantly higher survival rate compared with those without adjuvant therapy) were observed with ACT, but not with ACRT.

## Discussion

Despite significant progress in cancer therapeutics, there remains a lack of LSCC-specific treatment options, particularly for elderly patients with advanced diseases. For these patients, cisplatin-based postoperative adjuvant therapy remains the mainstream treatment option. However, the current TNM staging system is insufficient to serve as a treatment guideline due to its simplicity and heterogeneity. The formulation of individualized treatment regimens should take into account not only pathological stage, but also patient characteristics (e.g., age, comorbidities, individual will, performance status), medical assevssment, quality of resection [21] (grouped as complete, uncertain, and incomplete), and the balance of benefits and hazards [22, 23]. This large retrospective cohort study contributes significantly by developing and validating prognostic nomograms that incorporate the TNM staging system and other widely assessed prognostic variables for survival estimation. In addition, online decision tools that incorporate established nomograms for treatment optimization are expected to be integrated into clinical practice.

Nomogram A, B, and C were established and validated separately for patients with different treatment regimens. The higher c-indices indicated that these nomograms presented better discriminative capability than the TNM staging system both in the training and validation groups (all *P* < 0.05, Table 2). Similar superiority was also revealed by calculating time-dependent AUC in the training groups (Figure S4). Calibration curves (Figure S5) presented satisfactory consistency between actual observations and predicted CSS (Cancer-Specific Survival) probabilities in the training groups, indicating good repeatability of the nomograms. Similar outcomes (Figure S6) in the external validation groups proved that these nomograms could be widely used despite ethnic and geographical disparities.

A series of studies have demonstrated the feasibility of nomograms to predict survival for NSCLC [24–27]. However, the analysis of their impact on treatment decision-making was largely overlooked. To date, only a few studies have applied nomograms for treatment recommendation. Zhang et al. created nomograms to guide clinicians in choosing the optimal treatment strategy for locoregional nasopharyngeal cancer [28]. Jiang et al. developed a survival prediction model to guide individualized treatment recommendations for adjuvant chemotherapy in stage II/III gastric cancer [29]. Meanwhile, the application and dissemination of these prediction models have been hindered due to the lack of user-friendly interactive interfaces. The online decision tools created in this study may fill the void to some extent. By intuitively displaying the predicted LCSS of patients with different treatment regimens, the online tools could facilitate clinicians with decision-making, improving patients’ comprehension of the disease, medical teaching, experimental design, and so on. We displayed a hypothetical patient diagnosed with stage III A LSCC as example in Fig. 4. By comparing the CSS rates predicted by nomogram A, B, and C, we found that ACT might bring CSS benefits to this patient. However, no survival benefit was observed with ACRT. The ability of decision tools to predict the efficacy of AT is expected to be further used to guide related experimental design.

Several limitations existed in this study due to its retrospective nature, including but not limited to coding errors, selection bias (e.g., patients with missing clinical data were excluded, which may raise potential bias), and the absence of data in the SEER database (quality of surgery, targeted therapy information, immunotherapy information, radiotherapy dose, systemic therapy agents and so on). Moreover, performance status, a crucial variable influencing clinical decision-making, is also unavailable in the SEER database. It is worth mentioning that this study did not include patients who only received postoperative adjuvant radiotherapy due to its debated efficacy [22, 30, 31]. The developed nomograms need to be further verified by external data form other centers. Further efforts are warranted to collect prospective data and investigate the possibility of including other prognostic factors to improve the predictive performance. Conducting comparative trials between patients whose treatment decisions were influenced by the online tool and those whose decisions were not could provide more insights into the impact of the tool on clinical decision making. Finally, as stated in the disclaimer of the web tools, these risk prediction tools are the subject of ongoing research and will continue to be refined. Unless under the guidance of relevant researchers or clinicians, we do not recommend patients to independently use the current version of the web-based prediction model to avoid anxiety about the predicted prognosis.

## Conclusions

Prognostic nomograms that combine the TNM staging system with other prognostic variables were developed and validated in elderly patients with LSCC. The created online decision tools using these prognostic models are expected to be integrated into clinical practice for CSS estimation and postoperative treatment recommendation.

### Supplementary Information

**Additional file 1:** **Figure S1.** The forest plots showing the univariable Regression Analysis of variables associated with Cancer-Specific Survival. **Figure S2.** Multivariable adjusted hazard ratios for cancer-specific mortality according to age on a continuous scale. **Figure S3.** Survival curves depicting survival difference between groups stratified by different treatment regimens in the training group. **Figure S4.** Time-dependent area under the receiver operator characteristic curves (AUC) was calculated every 2 months from the 1st to the 60th month in the training group. **Figure S5.** The calibration curves of 1-, 3- and 5-year cancer-specific survival (CSS) based on nomogram prediction and actual observation in the training group. **Figure S6.** The calibration curves of 1-, 3- and 5-year cancer-specific survival (CSS) based on nomogram prediction and actual observation in the validation group. **Figure S7.** Decision Curve Analysis of nomograms for 1-, 3- and 5-year cancer-specific survival (CSS) in the training group. **Figure S8.** User-friendly online prognostic nomograms for cancer-specific survival estimation (only nomogram B was depicted).

## Data Availability

The information of validation cohort can be accessible upon reasonable request from the corresponding authors. E-mail addresses: Zhaojia0327@126.com (Jun Zhao), yangliqin9990@163.com (Li-qin Yang).

## Abbreviations

LSCC: Lung Squamous Cell Carcinoma
SEER: Surveillance, Epidemiology, and End Results
AUC: The area under the receiver operator characteristic curves
NSCLC: Non-small cell lung cancer
LUAD: Lung adenocarcinoma
PFS: Progression-free survival
OS: Overall survival
ICI: Immune checkpoint inhibitor
AT: Adjuvant therapy
ACT: Adjuvant chemotherapy
ACRT: Adjuvant chemoradiotherapy
LCSS: Lung Cancer-Specific Survival
CSS: Cancer-Specific Survival
IQR: Interquartile range
HR: Hazard ratio
C-index: The concordance index
ROC: Receiver operating characteristic
DCA: Decision curve analysis
CI: Confidence interval
