# Supplementary material for: Online decision tools for personalized survival prediction and treatment optimization in elderly patients with lung squamous cell carcinoma: a retrospective cohort study
Source: BMC Cancer. 2023 Sep 29;23:920. doi: 10.1186/s12885-023-11309-z (PMC10542697; doi:10.1186/s12885-023-11309-z)
Supplement: Supplementary file 1 — Additional file 1: Figure S1. The forest plots showing the univariable Regression Analysis of variables associated with Cancer-Specific Survival. Figure S2. Multivariable adjusted hazard ratios for cancer-specific mortality according to age on a continuous scale. Figure S3. Survival curves depicting survival difference between groups stratified by different treatment regimens in the training group. Figure S4. Time-dependent area under the receiver operator characteristic curves (AUC) was calculated every 2 months from the 1st to the 60th month in the training group. Figure S5. The calibration curves of 1-, 3- and 5-year cancer-specific survival (CSS) based on nomogram prediction and actual observation in the training group. Figure S6. The calibration curves of 1-, 3- and 5-year cancer-specific survival (CSS) based on nomogram prediction and actual observation in the validation group. Figure S7. Decision Curve Analysis of nomograms for 1-, 3- and 5-year cancer-specific survival (CSS) in the training group. Figure S8. User-friendly online prognostic nomograms for cancer-specific survival estimation (only nomogram B was depicted). [file 12885_2023_11309_MOESM1_ESM.pdf]

Supplementary Online Content

Figure S1 The forest plots showing the univariable Regression Analysis of variables associated with Cancer-Specific Survival. (Page 2)

Figure S2 Multivariable adjusted hazard ratios for cancer-specific mortality according to age on a continuous scale. (Page 3)

Figure S3 Survival curves depicting survival difference between groups stratified by different treatment regimens in the training group. (Page 4)

Figure S4 Time-dependent area under the receiver operator characteristic curves (AUC) was calculated every 2 months from the 1st to the 60th month in the training group. (Page 5)

Figure S5 The calibration curves of 1-, 3- and 5-year cancer-specific survival (CSS) based on nomogram prediction and actual observation in the training group. (Page 6)

Figure S6 The calibration curves of 1-, 3- and 5-year cancer-specific survival (CSS) based on nomogram prediction and actual observation in the validation group. (Page 7)

Figure S7 Decision Curve Analysis of nomograms for 1-, 3- and 5-year cancer-specific survival (CSS) in the training group. (Page 8)

Figure S8 User-friendly online prognostic nomograms for cancer-specific survival estimation (only nomogram B was depicted). (Page 9)

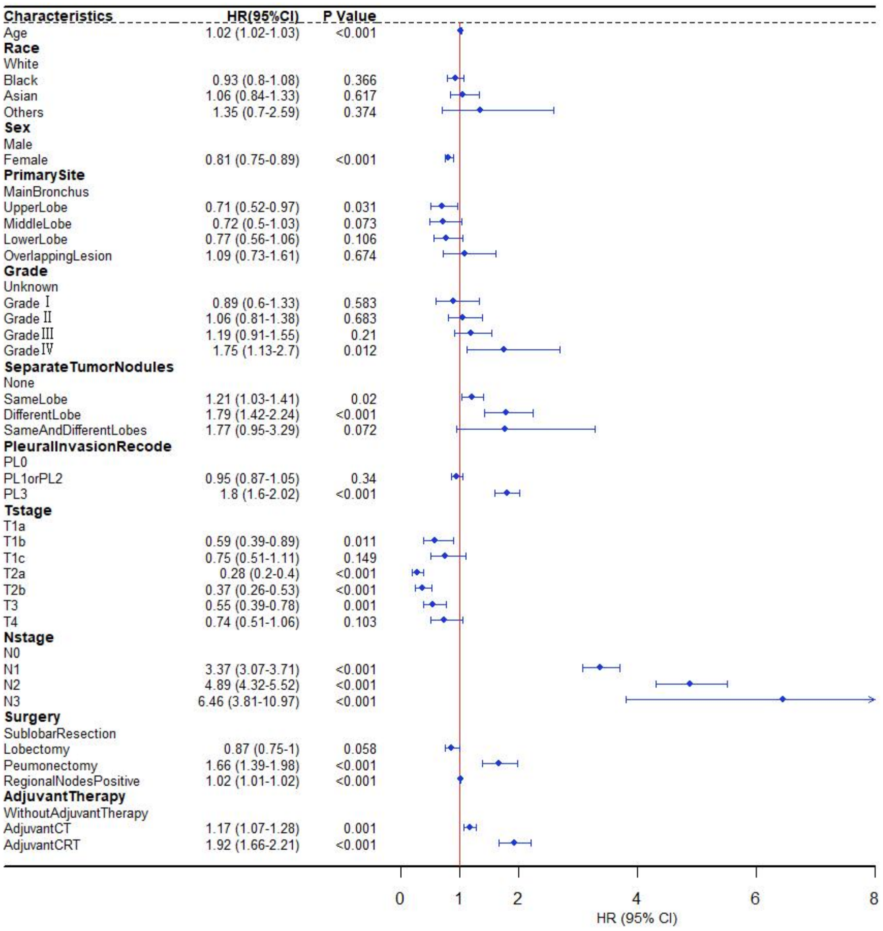

Figure S1 The forest plots showing the univariable Regression Analysis of variables associated with Lung Cancer-Specific Survival. Grade I, well differentiated; Grade II, moderately differentiated; Grade III, poorly differentiated; Grade IV, undifferentiated; PL0, tumor does not completely traverse the elastic layer of pleura; PL1 or PL2, invasion of visceral pleura present; PL3, tumor invades into or through the parietal pleura or chest wall; CT, chemotherapy; CRT, chemoradiotherapy

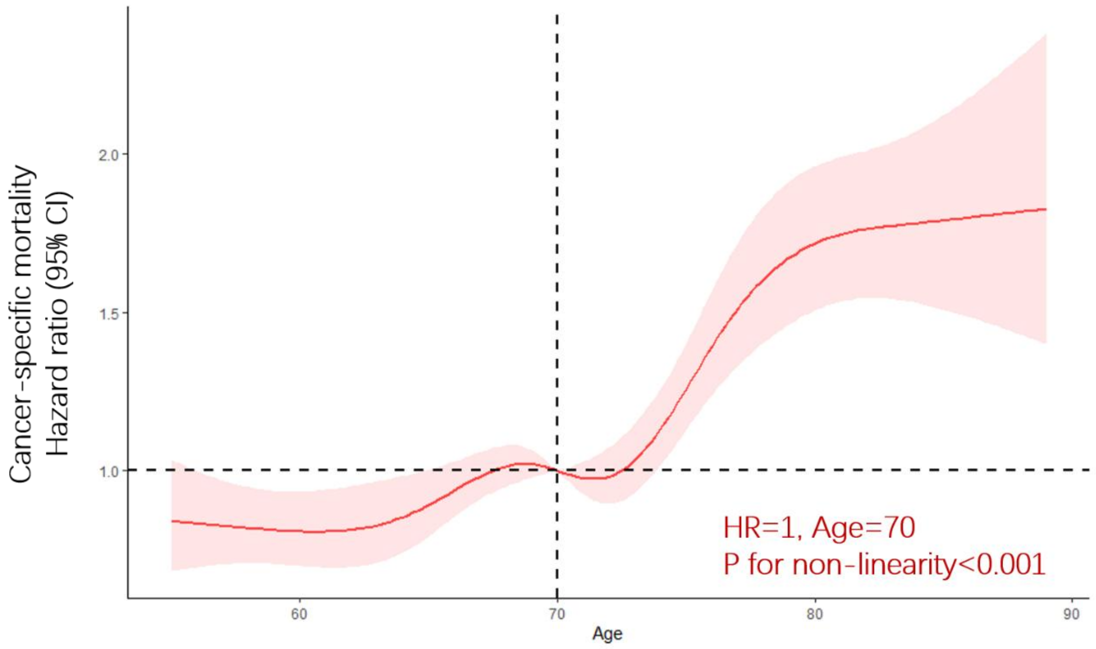

Figure S2 Multivariable adjusted hazard ratios for lung cancer-specific mortality according to age on a continuous scale. Multivariable adjusted hazard ratios are indicated by solid red lines and 95% CIs by shaded areas. The optimal number of knots was identified as 6, which placed at 5th, 35th, 65th, and 95th centiles of age. Analyses were adjusted for race, sex, grade, separate tumor nodules, pleural invasion record, T stage, N stage, Surgery, regional nodes positive, and adjuvant therapy.

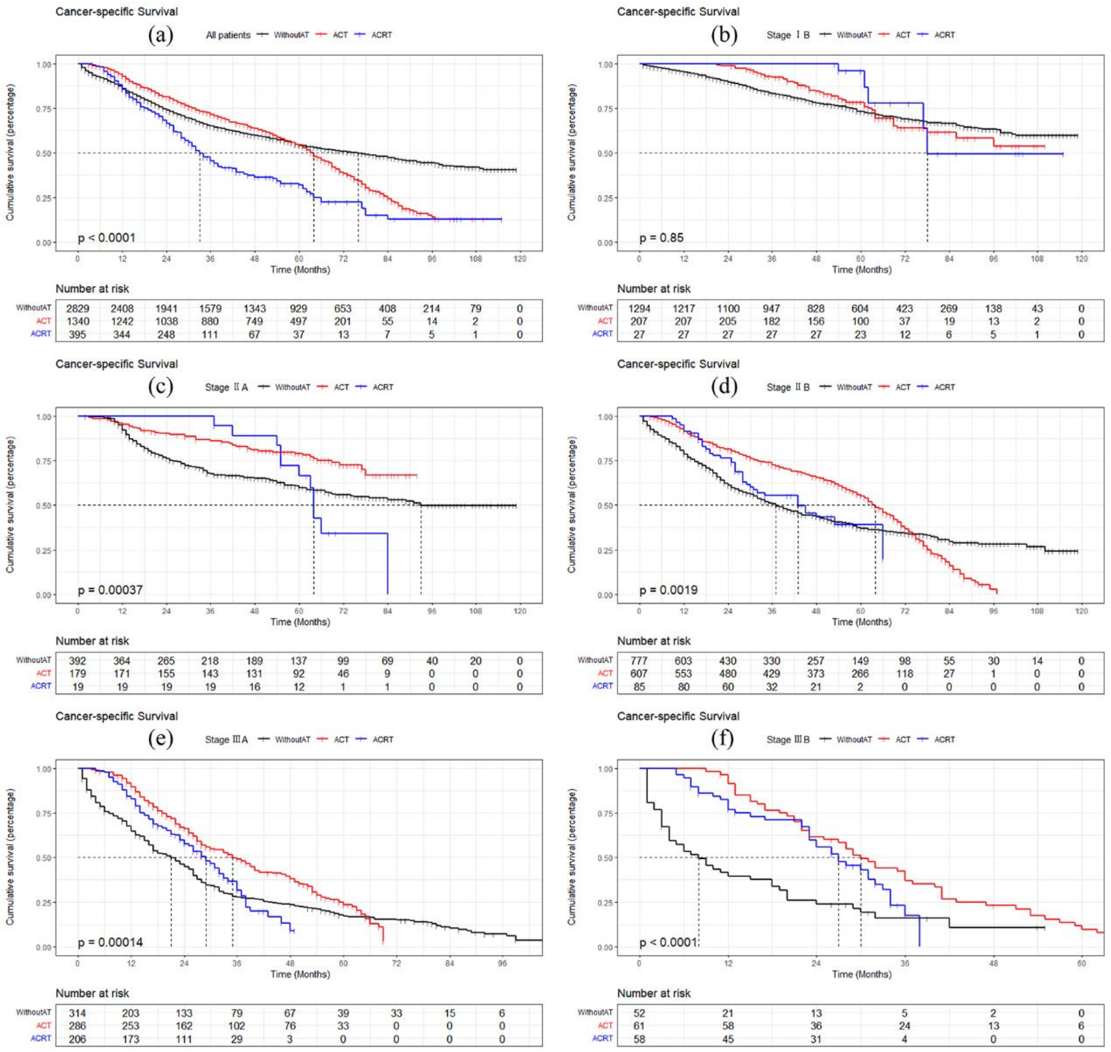

Figure S3 Survival curves depicting survival difference between groups stratified by different treatment regimens in the training group. Without AT, without adjuvant therapy; ACT, adjuvant chemotherapy; ACRT, adjuvant chemoradiotherapy

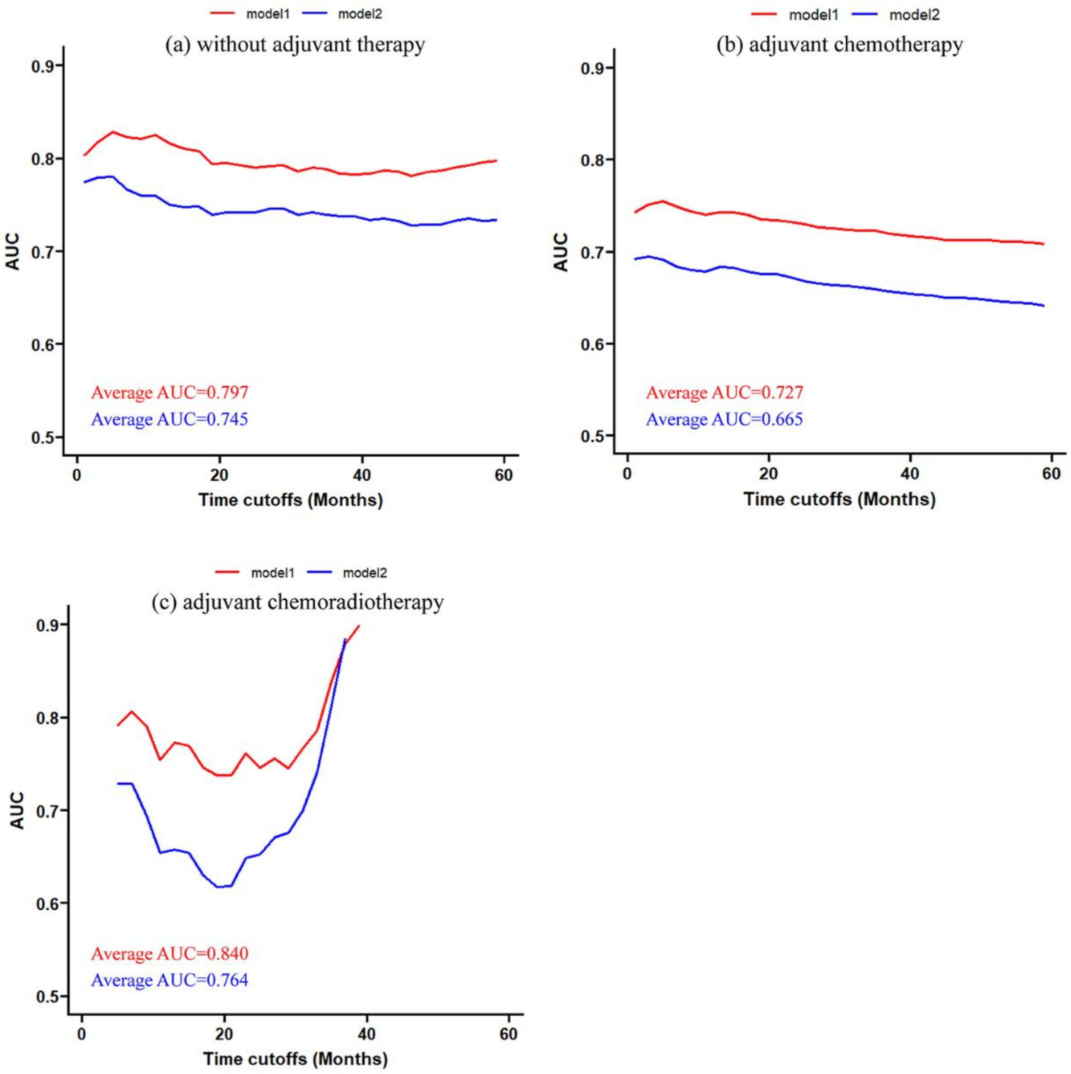

Figure S4 Time-dependent area under the receiver operator characteristic curves (AUC) was calculated every 2 months from the 1st to the 60th month in the training group. Average AUC was calculated by averaging the 30 AUC values. Patients were stratified by (a) without adjuvant therapy, (b) adjuvant chemotherapy, and (c) adjuvant chemoradiotherapy. Red, nomograms developed in this study; Blue, AJCC 8th TNM staging system

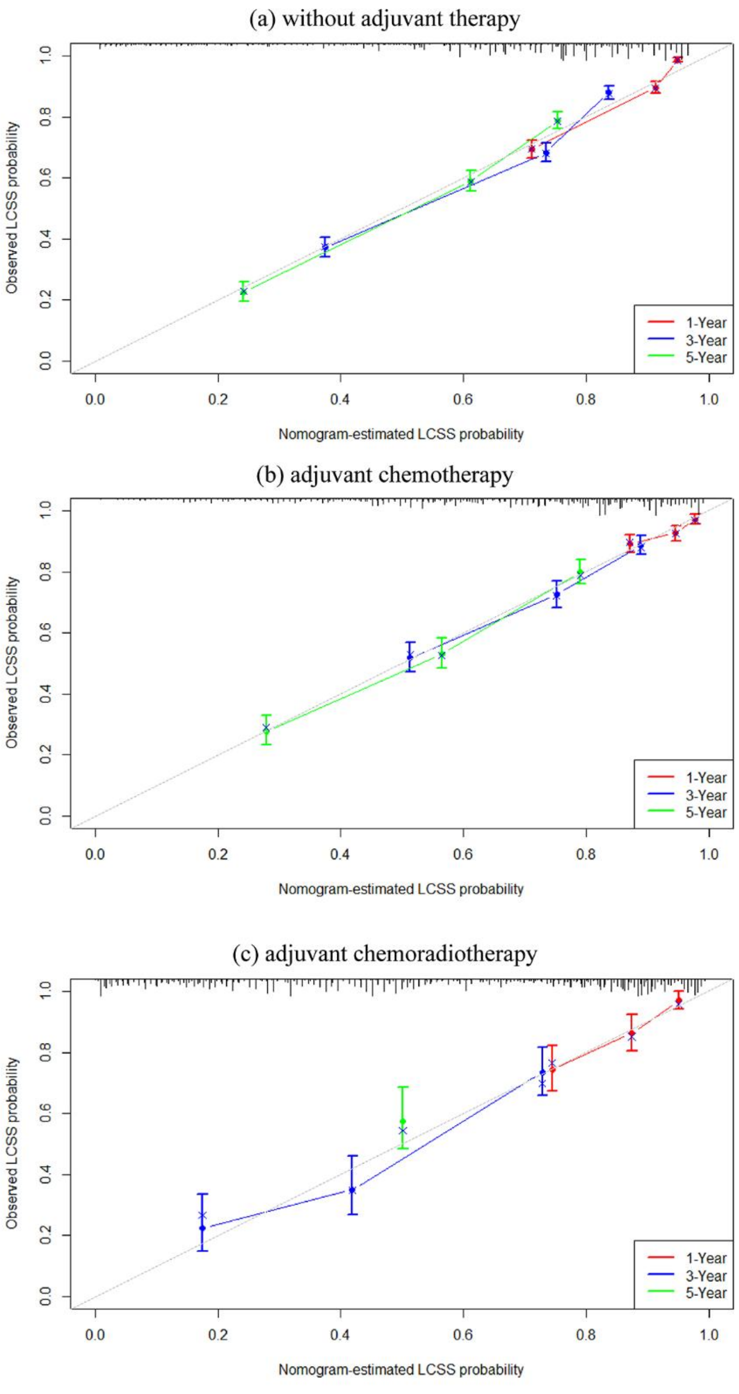

Figure S5 The calibration curves of 1-, 3- and 5-year cancer-specific survival (CSS) based on nomogram prediction and actual observation in the training group. Patients were stratified by (a) without adjuvant therapy, (b) adjuvant chemotherapy, and (c) adjuvant chemoradiotherapy. Each point in the plot refers to a group of patients, with the nomogram predicted probability of survival shown on x-axis and actual survival proportion shown on y-axis. A standard curve of the calibration plot is a straight line through the origin of the axes with a slope of 1. when the prediction line falls on the 45-degree diagonal more, the model is more accurate. Distributions of predicted survival probabilities are plotted at the top. Error bars represent 95% confidence intervals.

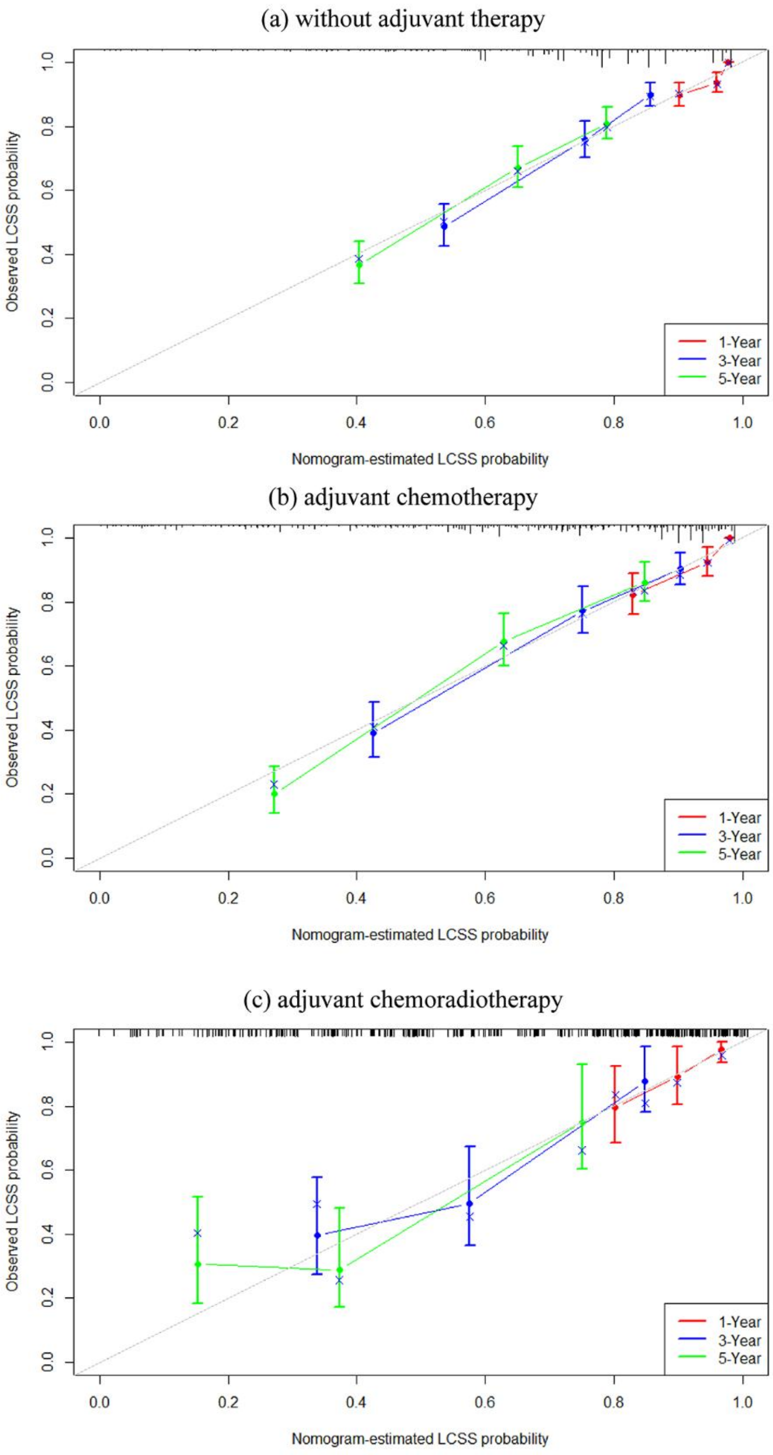

Figure S6 The calibration curves of 1-, 3- and 5-year cancer-specific survival (CSS) based on nomogram prediction and actual observation in the validation group. Patients were stratified by (a) without adjuvant therapy, (b) adjuvant chemotherapy, and (c) adjuvant chemoradiotherapy. Each point in the plot refers to a group of patients, with the nomogram predicted probability of survival shown on x-axis and actual survival proportion shown on y-axis. A standard curve of the calibration plot is a straight line through the origin of the axes with a slope of 1. when the prediction line falls on the 45-degree diagonal more, the model is more accurate. Distributions of predicted survival probabilities are plotted at the top. Error bars represent 95% confidence intervals.

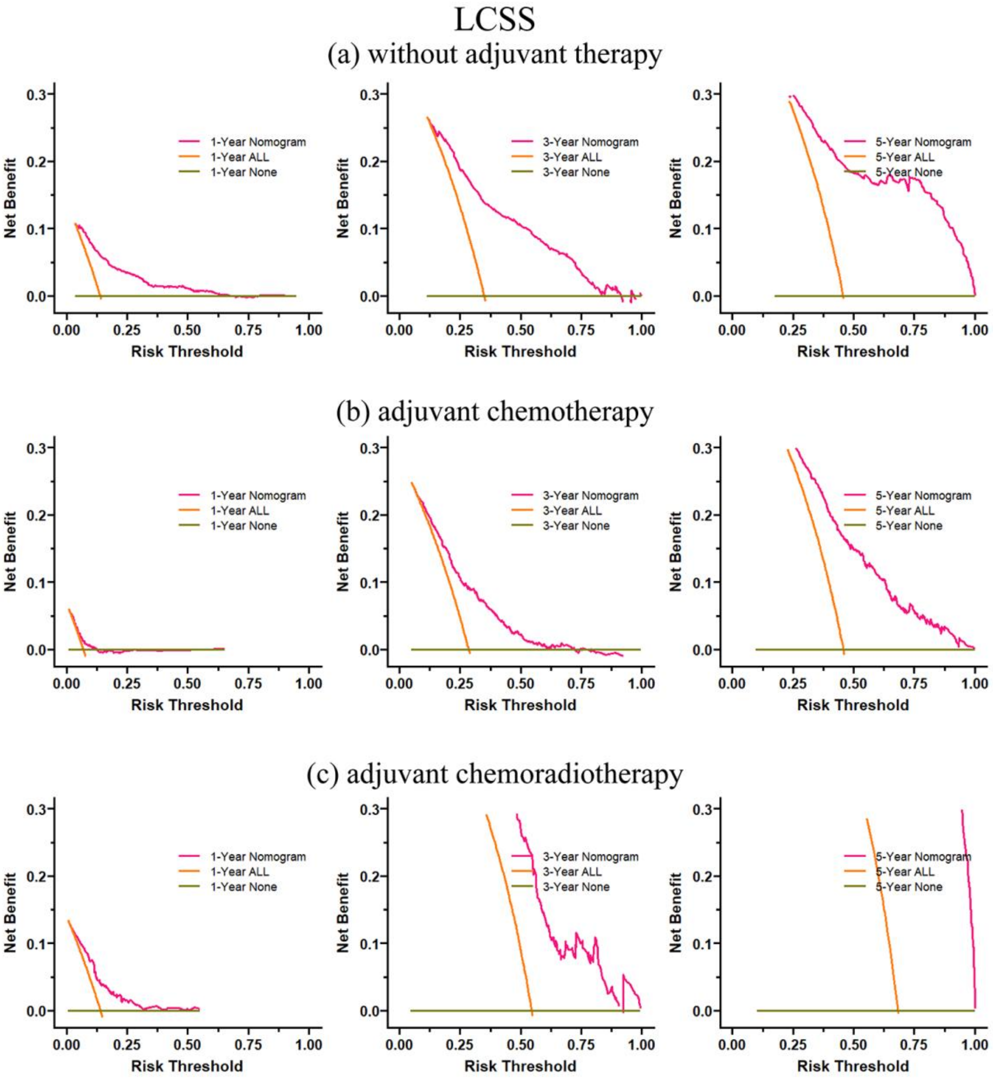

Figure S7 Decision Curve Analysis (DCA) of nomograms for 1-, 3- and 5-year Lung Cancer-Specific Survival (LCSS) in the training group. Patients were stratified by (a) without adjuvant therapy, (b) adjuvant chemotherapy, and (c) adjuvant chemoradiotherapy. DCA curves evaluate nomograms from the perspective of clinical benefit and scope of clinical benefits. The y-axis represents the net benefit. The x-axis represents the predicted LCSS probability. The orange line represents the condition that all patients survive during the follow-up period, while the dark green line represents the condition that all patients died during the follow-up period. The red line represents the net benefit using established nomograms at different risk thresholds.

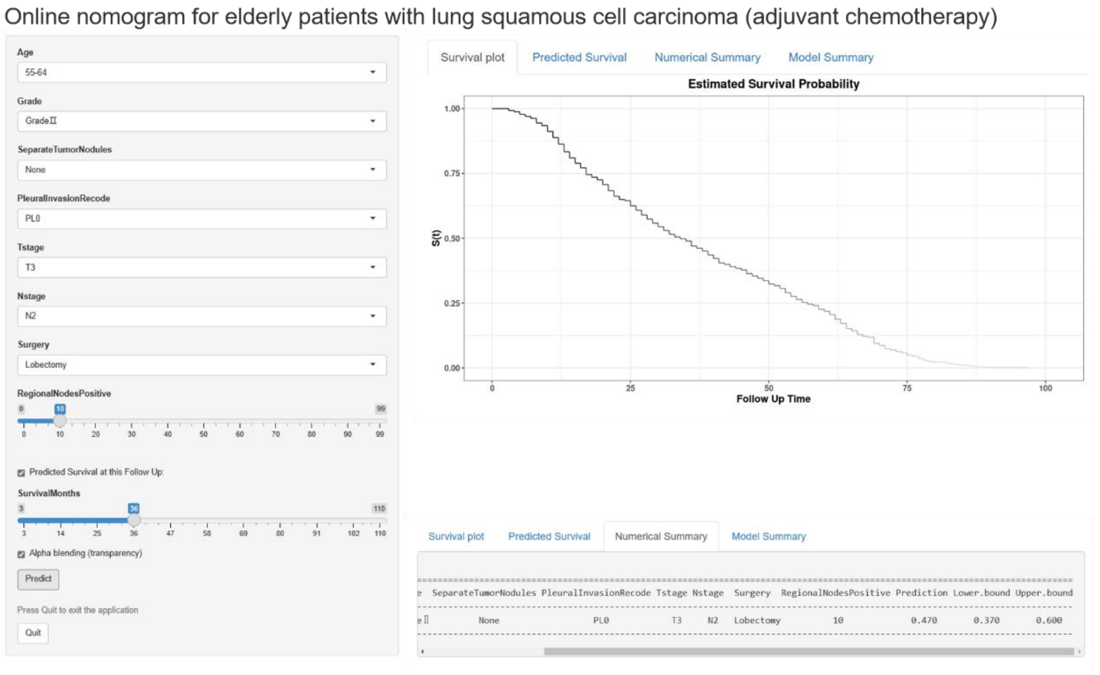

Figure S8 User-friendly online prognostic nomograms for cancer-specific survival estimation (only nomogram B was depicted). This example shows the estimated Kaplan-Meier curve (top right) and the predicted survival probability at 60 months (bottom right) of a hypothetical patient. The detailed characteristics of this patient were shown in the left-hand of the diagram.
